# Satisfaction with urban rural sports integration at the county level in China and its determinants

**DOI:** 10.3389/fpubh.2026.1821897

**Published:** 2026-04-30

**Authors:** Xiangjun Yang, Xiujin Guo

**Affiliations:** 1 Taizhou University, College of Teacher Education, Taizhou, China; 2 Nanjing Sport Institute, School of Sport Industry and Leisure, Nanjing, China

**Keywords:** county level sport governance, digital linkage, integration satisfaction, multilevel linear model, social sports instructor, urban rural sports integration

## Abstract

**Objective:**

Against the backdrop of continued expansion in the scale of county-level public sport service provision in China without commensurate improvement in residents' user experience, and with persistent disparities in perceived gains between urban and rural areas, this study treats satisfaction with urban–rural sports integration as the outcome variable. It assesses the level of satisfaction and examines the roles of digital linkage and village-level supply capacity in shaping satisfaction differences, while quantifying the contribution of village context.

**Methods:**

A nationally stratified survey was conducted (*n* = 3,595). Mean satisfaction scores were computed from an 18-item, 5-point Likert scale, followed by reliability testing and principal component extraction. After controlling for individual characteristics and sport participation, a random-intercept multilevel linear model (individuals nested within villages) was used to estimate determinants. Robustness checks included replacing the mean satisfaction score with the first principal component score, estimating an OLS model with cluster-robust standard errors as an alternative to multilevel modeling, and conducting sensitivity analyses using different strategies to handle uncertain responses on the digital linkage measure.

**Results:**

The overall satisfaction score was 3.37 (moderately high), with the lowest ratings related to the fitness e-map. The scale showed high internal consistency (α = 0.9736), and the first principal component explained 69.09% of the total variance. Village-level heterogeneity was substantial (ICC = 0.2114). Digital linkage was positively associated with satisfaction (β = 0.1244, *p* < 0.001), though the effect attenuated after accounting for village capacity. The presence of social sports instructors significantly increased satisfaction (β = 0.1780, *p* < 0.001), whereas volunteer services were negatively associated with satisfaction (β = −0.3134, *p* < 0.001). A cross-domain leadership background was not statistically significant. Robustness checks yielded consistent directions.

**Conclusions:**

Residents' perceived gains are driven primarily by village-level organization and human resource provision. Digital platforms cannot substitute for offline service delivery. County-level governance should shift from expanding provision to improving usability, prioritizing the development of social sports instructor teams and organizational operations, enhancing the quality and fit of volunteer services, and promoting routine updating and user-friendly design of the fitness e-map.

## 1 Introduction

Counties constitute the primary arena in which public sport services are delivered and are also among the spatial units where urban and rural factors circulate most intensively (1). Whether urban rural sports integration translates into residents perceived gains is ultimately tested through everyday service provision and user experience at the county level. With the sustained rollout of the National Fitness strategy in recent years, official statistics from the General Administration of Sport of China indicate that in 2024 the number of sport venues nationwide reached 4.8417 million, with a total area of 4.23 billion square meters and approximately 3.0 square meters per capita, suggesting that provision has achieved a substantial scale base (2). The National Fitness Plan for 2021 to 2025 calls for improving the public service system and advancing the digital upgrading of public sport facilities, emphasizing greater accessibility and efficiency (3). Yet growth in provision does not necessarily translate into better user experience, and urban rural disparities persist. According to the 2020 National Fitness Activity Survey released by the National Physical Fitness Monitoring Center, 67.5 percent of residents aged 7 and above engaged in physical exercise at least once per week, compared with 70.4 percent in urban areas and 63.1 percent in rural areas. Frontline service capacity has also expanded (4). By the end of 2024, the cumulative number of social sports instructors nationwide was about 3.71 million, or roughly 2.63 per thousand people, providing institutional support for evidence based fitness guidance (5). Taken together, county level sport governance is shifting from whether services exist to whether they are usable, and evaluation systems need to align more closely with resident experience and the actual functioning of the service chain (6).

Existing work on urban rural sport services has mainly focused on three strands: public sport service provision and equity, participation and facility accessibility, and satisfaction with service quality (7–9). However, direct evidence on how county level urban rural sports integration is perceived remains limited. Integration satisfaction is seldom used as a comprehensive performance outcome, as many studies emphasize provision scale or participation rates (10, 11). In addition, village level clustering and contextual heterogeneity are rarely modeled, and digital linkage and grassroots human resources are seldom examined jointly (10).

Accordingly, this study treats satisfaction with county level urban rural sports integration as the primary outcome. We describe the level and structure of the satisfaction scale, and then estimate random intercept multilevel models with individuals nested within villages, adjusting for gender, age, education, occupation, income, and sport participation. We assess the independent associations of digital linkage and village capacity with satisfaction and derive governance implications for resource coordination and service usability.

## 2 Methods

This study aims to systematically assess, from the perspective of rural residents, the level of satisfaction with county level urban rural sports integration in China and to identify its determinants. Focusing on key links in the integration of county level public sport services and the flow of relevant factors, the analysis examines the roles of digital linkage such as incorporating village sport facilities into the county fitness electronic map, organizational and resource inputs from county seats, and grassroots supply capacity including village sport organizations, social sports instructors, and volunteer services. Multilevel modeling is used to identify the main drivers of satisfaction with urban rural sports integration and to provide empirical evidence for optimizing resource allocation and improving integration governance at the county level.

### 2.1 Data sources and sample

To describe county level urban rural sports integration at the national level, we conducted a stratified survey using a questionnaire administered through a mix of household visits and centralized sessions. We followed the National Bureau of Statistics regional classification and defined four strata: Eastern, Central, Western, and Northeastern China. We sampled Guangdong, Zhejiang, and Jiangsu in the Eastern stratum; Henan, Anhui, and Hunan in the Central stratum; Chongqing, Shaanxi, and Guizhou in the Western stratum; and Heilongjiang in the Northeastern stratum. Within each selected province, prefecture level cities were sampled around the provincial median GDP, followed by stepwise sampling of counties or districts, townships or subdistricts, and villages. Two representative villages were selected within each township. Data were collected using the Questionnaire for Village Residents on Urban Rural Sports Integration Development in China (12). Trained enumerators administered the survey with support from grassroots organizations, and responses were anonymous to reduce social desirability bias. This paper analyzes the villager questionnaire. A total of 5,000 questionnaires were distributed, and 3,595 valid responses were included in the final analytic sample. The survey recorded province, city, county, township, and village identifiers, enabling multilevel analyses with individuals nested within villages and counties. The spatial distribution of the four macro regions and the sampled provinces is shown in Supplementary Figure S1.

### 2.2 Variables and measurement

#### 2.2.1 Dependent variable: satisfaction with urban rural sports integration

Satisfaction with urban rural sports integration is the primary dependent variable. It was measured using 18 items from Module D1, each rated on a 5 point Likert scale where 1 indicates very dissatisfied and 5 indicates very satisfied. The items cover convenience of sport participation, fiscal and social support, facility integration and digital development, provision of training, events and publicity, protection of traditional sport, and sport plus tourism development. For the main analyses, an overall satisfaction index was constructed as the row mean of the 18 items and denoted Satis_mean. To ensure indicator stability, the mean was computed only when a respondent provided valid answers for at least a pre-specified proportion of items; otherwise, the index was coded as missing. To test sensitivity to outcome construction, exploratory factor analysis and principal component analysis were used to examine the item structure, and either subscales or the first principal component score was used as an alternative outcome in robustness checks. Scale reliability was assessed using Cronbach alpha, and item total correlations were reported to evaluate consistency with the overall construct and item discrimination, thereby providing a sound measurement basis for subsequent modeling.

#### 2.2.2 Key independent variables: county seat inputs and linkage intensity

County seat inputs and linkage intensity capture the extent to which resources, organizations, and information flow from the county level into rural areas. Organizational input reflects the breadth of county based sport organizations entering villages to deliver events and activities. It was constructed as a count based on the reported types of organizations from the county seat, excluding response options that provide little substantive information such as do not know, no county organizations, or not yet held. Financial input represents the breadth of linkage between village event funding and county level funding sources and was measured as a count of selected county funding channels, again excluding do not know and no county funding channels. Participation input captures the coverage of county seat personnel involved in village events and was measured as a count of selected categories of county participants, excluding do not know and no county participants. Digital linkage focuses on whether village sport facilities are included in the county fitness electronic map and the usability of that inclusion. In the main models, this variable was retained as a four category measure, namely included and easy to search, included but difficult to search, not included, and do not know. In robustness analyses, it was collapsed into a binary indicator of included vs. not included to assess sensitivity to alternative coding schemes.

#### 2.2.3 Key independent variables: village organizational capacity and human resource supply

Village organizational capacity and human resource supply reflect the organizational foundation of grassroots sport governance and the level of service provision (13, 14). The existence of a village sport organization was coded as a binary indicator using reverse coding based on whether the respondent selected the option indicating no sport organization. The cross domain or mobile background of organizational leaders was used to indicate the village ability to connect with county seat social networks. Background characteristics such as out migration for work, return after retirement, secondment as a village cadre or volunteer, and urban residence combined with work in the village were treated as cross domain linkage attributes. This construct was operationalized either as a binary indicator for having any such attribute or as a count of attributes, depending on model specifications. The supply of social sports instructors was measured as a binary indicator of whether the village had social sports instructors, and instructor background information could be summarized as a count for extended analyses. Volunteer service provision was classified by perceived quality into good service, average service, and no service. It was entered into models either as an ordered variable coded 2, 1, and 0 to represent supply intensity or as a three category variable for robustness checks.

### 2.3 Variables and measurement

To reduce confounding from individual differences, control variables covered demographic and socioeconomic characteristics as well as sport participation behavior (15). Demographic and socioeconomic controls included gender, age group, educational attainment, occupational status, and household income, capturing individual resource endowments and social stratification. Sport participation behavior was measured using weekly participation frequency and duration per session to reflect routine exercise habits. In the main models, weekly participation frequency was included as the participation control. Duration per session was used only in robustness analyses among respondents who reported engaging in sport activity, because it is meaningful only for participants. If duration is to be incorporated in the full sample, a two part specification separating participation status from duration among participants is recommended to avoid treating structural missingness as low duration and thereby introducing bias.

### 2.4 Statistical analysis

Data cleaning and statistical analyses were conducted in Python 3. Sample characteristics and exercise behaviors were summarized using descriptive statistics. For categorical variables, frequencies and percentages were reported. Because session duration is meaningful only among those who exercised, duration statistics were computed using the participating subsample as the denominator with the effective sample size explicitly reported. Satisfaction with urban rural sports integration was measured using the 18 item 5 point scale, and the primary outcome was the row mean of items subject to a minimum number of valid responses to ensure stability. Internal consistency was assessed using Cronbach alpha, and item means and standard deviations were reported along with item total correlations to evaluate alignment with the overall construct. Suitability for dimensionality reduction was evaluated using the Kaiser Meyer Olkin measure and Bartlett test of sphericity. When criteria were met, principal component analysis was performed and eigenvalues, explained variance, and the loading matrix were reported. The first principal component score was used as an alternative satisfaction indicator to test sensitivity to outcome construction. Data processing and tabulation relied on pandas, KMO and Bartlett tests were implemented using factor_analyzer, and PCA was conducted with the PCA module in sklearn.

Given the nested structure of respondents within villages, the main analyses used a random intercept multilevel linear model with village level random intercepts to account for within village correlation. Parameters were estimated using restricted maximum likelihood. Models were built in a nested stepwise manner. An unconditional model was first estimated to compute the intraclass correlation coefficient and quantify the share of variance attributable to villages. A control model then added demographic and socioeconomic characteristics and weekly participation frequency. An extended model incorporated county seat inputs and digital linkage indicators. The final model further included village organizational capacity and human resource supply variables, including cross domain leader characteristics, the presence of social sports instructors, and volunteer service level. Fixed effect coefficients, standard errors, and significance levels were reported, along with random effect variance components and fit indices to compare improvements across models. Robustness checks covered three sets of specifications: reestimating models using the first principal component score instead of the mean satisfaction index, using ordinary least squares with cluster robust standard errors instead of multilevel modeling, and conducting alternative coding strategies for do not know responses on the digital linkage measure, including treating them as missing or adding an indicator variable. All tests were two sided, with significance levels set at 0.05, 0.01, and 0.001.

## 3 Results

### 3.1 Sample characteristics and sport participation

The study surveyed 3,595 rural residents, with a nearly balanced gender distribution, 50.21 percent male and 49.79 percent female. The age profile was dominated by young and middle aged adults. Respondents aged 31 to 45 accounted for the largest share at 33.69 percent, followed by those aged 19 to 30 at 27.01 percent and those aged 46 to 59 at 20.08 percent. Respondents aged 18 or younger and 60 or older accounted for 8.18 percent and 8.26 percent, respectively. In terms of education, junior high school was the most common level at 31.38 percent. Senior high school or vocational secondary school and master's degree or above accounted for 21.25 percent and 21.11 percent, respectively. Primary school or below accounted for 13.71 percent, and junior college or bachelor's degree accounted for 10.26 percent. Occupationally, self employment was most prevalent at 32.49 percent, followed by employment in public institutions or enterprises at 24.06 percent. Retired or unemployed respondents accounted for 14.30 percent, migrant workers for 8.62 percent, and farmers for 2.92 percent. Regarding sport participation, exercising one to two times per week was most common at 38.19 percent, while the shares reporting no participation and occasional participation defined as one to three times per month were similar at 20.81 percent and 20.56 percent. Among respondents with valid duration data, *n* equals 2,847, a single exercise session most often lasted 30 to 60 min at 40.36 percent, and 21.64 percent reported sessions shorter than 30 min. Table 1 summarizes the sample profile and sport participation patterns.

**Table 1:** Sample characteristics of respondents and sport participation patterns.

Variable	Category	Frequency (*n*)	Percentage (%)
Gender	Male	1,805	50.21
	Female	1,790	49.79
Age	≤ 18 years	294	8.18
	19–30 years	971	27.01
	31–45 years	1,211	33.69
	46–59 years	722	20.08
	≥60 years	297	8.26
Education	Primary school or below	493	13.71
	Junior high school	1,128	31.38
	Senior high/technical secondary school	764	21.25
	Junior college/bachelor's degree	369	10.26
	Master's degree or above	759	21.11
Occupation	Farming	105	2.92
	Wage employment	310	8.62
	Self-employed	1,168	32.49
	Enterprises/public institutions	865	24.06
	Retired/unemployed	514	14.3
	Other	455	12.66
Exercise frequency	Never	748	20.81
	Occasionally (1–3 times/month)	739	20.56
	Sometimes (1–2 times/week)	1,373	38.19
Exercise duration (valid *n* = 2,847)	<30 min	616	21.64
	30–60 min	1,149	40.36
	61–90 min	243	8.54
	91–120 min	111	3.9

### 3.2 Item characteristics and reliability of the satisfaction scale

**Figure 1:**
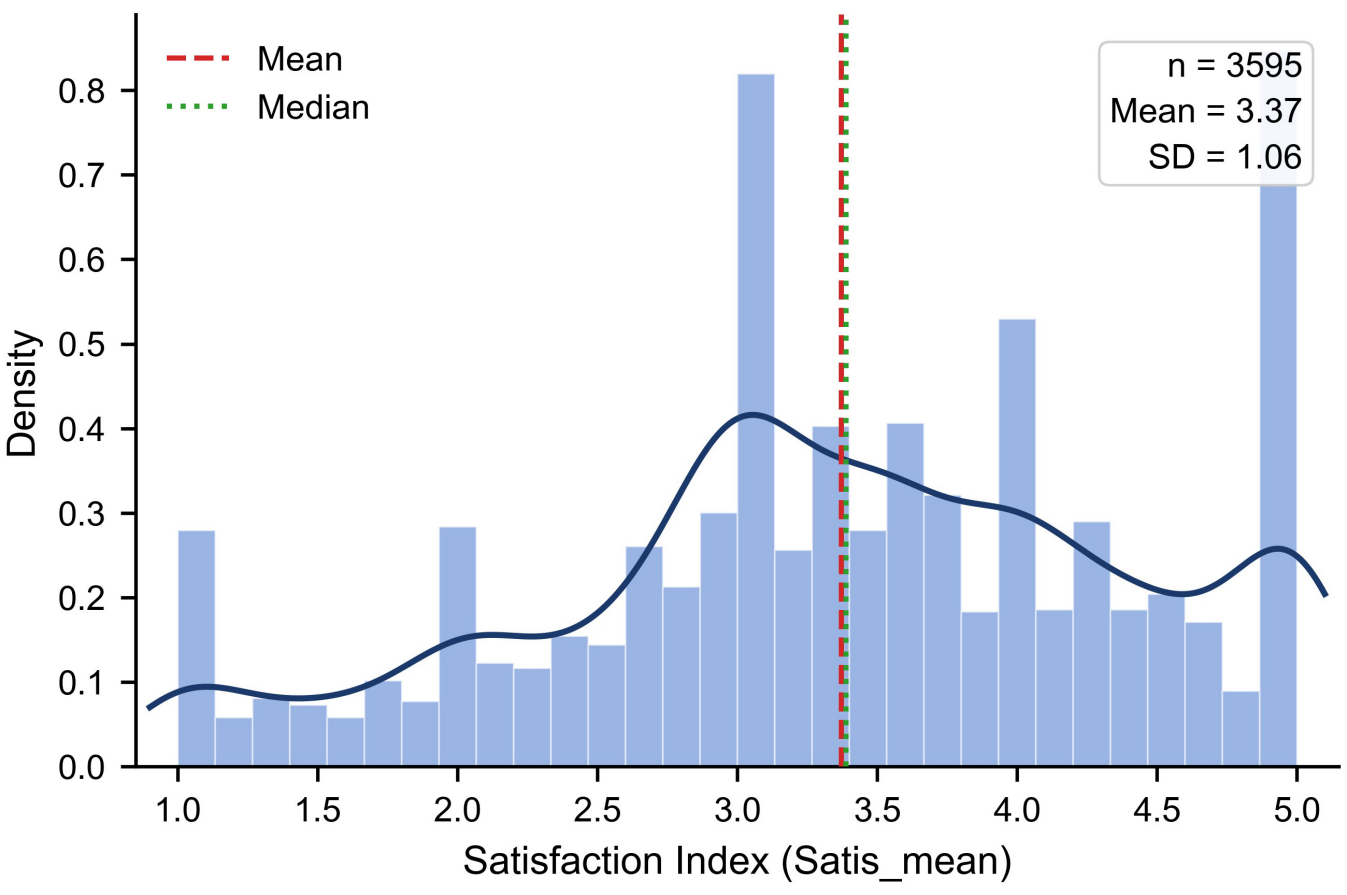
Distribution of the overall county level urban rural sports integration satisfaction score.

**Figure 2:**
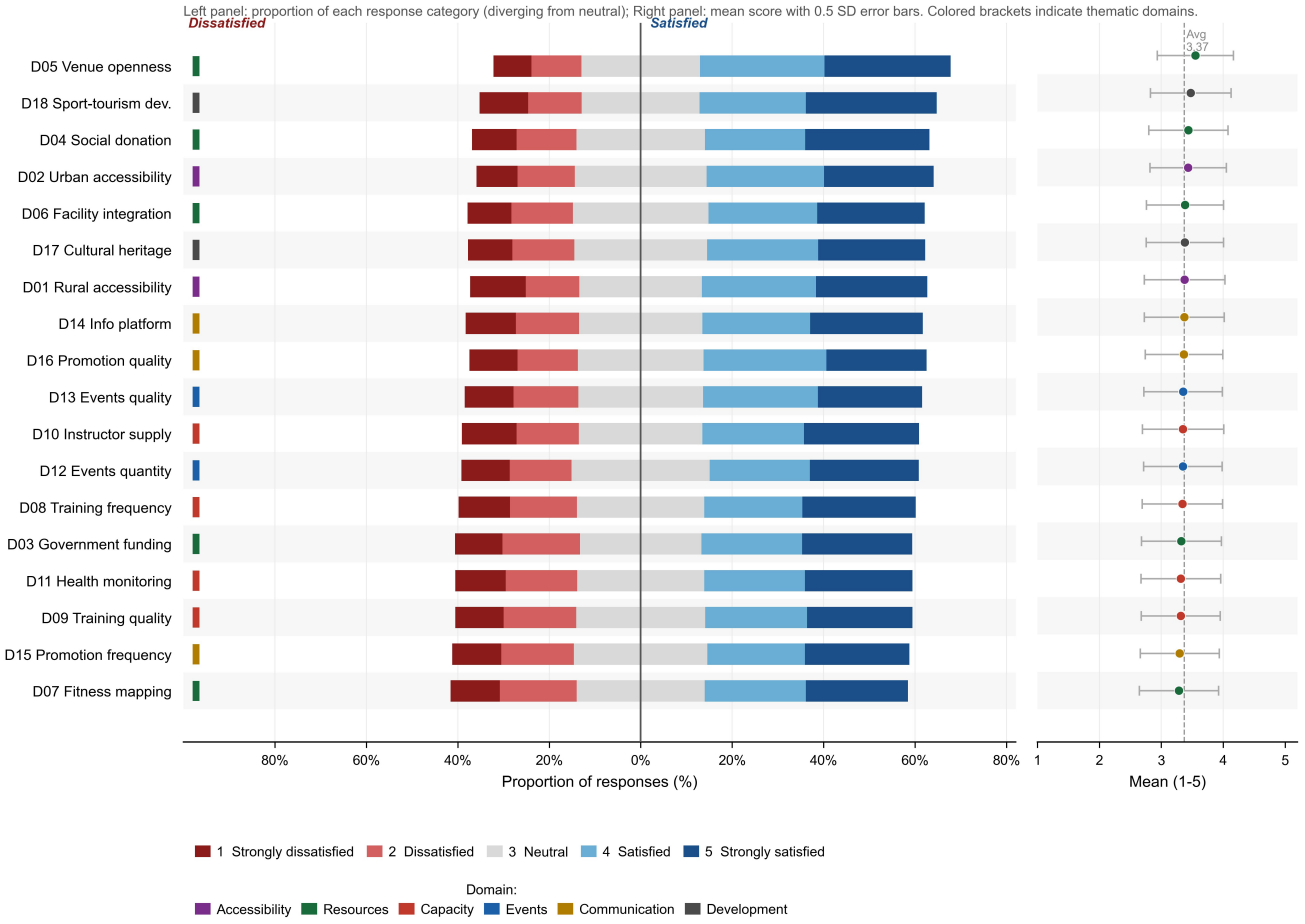
Likert response profile for the 18 satisfaction items and identification of low rated domains.

The overall mean of the satisfaction scale was 3.37 as shown in Figure 1, and item means ranged from 3.28 to 3.55 as shown in Figure 2, indicating a moderately high overall evaluation of county level urban rural sports integration as reported in Table 2. At the item level, the highest rated item was free or low fee access to public sport venues and facilities in the county seat, with a mean of 3.55 and standard deviation of 1.23. It was followed by development of rural sport tourism and related industries at 3.47 with standard deviation 1.30, and donations from county seat residents or enterprises to support rural sport development at 3.44 with standard deviation 1.28. These patterns suggest relatively positive perceptions of facility accessibility and social support. By contrast, the county fitness facility electronic map received the lowest rating at 3.28 with standard deviation 1.28, indicating room for improvement in the provision and usability of digital services. Internal consistency was high, with Cronbach alpha of 0.9736. Item total correlations ranged from 0.727 to 0.850, showing strong alignment between individual items and the overall construct and no evidence of weakly related items. Overall, the scale demonstrates high reliability and good item discrimination and is suitable for constructing a composite satisfaction indicator for subsequent modeling.

**Table 2:** Descriptive statistics and item–total correlations for the urban–rural sports integration satisfaction scale (D01–D18).

Item	English label	Mean	SD	Mean ±SD	Item–total correlation (*r*)
D01	Convenience for urban residents to participate in rural events	3.3755	1.2988	3.38 ± 1.30	0.7556
D02	Convenience for rural residents to participate in county-town events	3.4325	1.2303	3.43 ± 1.23	0.7724
D03	Fiscal support from county-level sports finance	3.3241	1.2899	3.32 ± 1.29	0.8003
D04	Social donations from county-town residents/enterprises	3.437	1.2789	3.44 ± 1.28	0.8037
D05	Openness of county-town public sports facilities (free/low-fee)	3.5497	1.2314	3.55 ± 1.23	0.727
D06	Integrated layout of urban–rural sports facilities within the county	3.3819	1.2457	3.38 ± 1.25	0.8151
D07	Development of a county-wide fitness facility e-map	3.2843	1.2775	3.28 ± 1.28	0.8053
D08	Frequency of sports training provided in rural areas	3.3394	1.2994	3.34 ± 1.30	0.8341
D09	Quality of sports training provided in rural areas	3.3132	1.2762	3.31 ± 1.28	0.8272
D10	Number of sport instructors in the village	3.3505	1.3118	3.35 ± 1.31	0.8244
D11	Coverage of rural national fitness monitoring stations	3.3135	1.2886	3.31 ± 1.29	0.8173
D12	Number of sports events held in rural areas	3.3483	1.2679	3.35 ± 1.27	0.8416
D13	Quality of sports events held in rural areas	3.3513	1.2679	3.35 ± 1.27	0.8499
D14	Unified information exchange platform for urban–rural sports events/activities	3.3705	1.2893	3.37 ± 1.29	0.8214
D15	Frequency of sports publicity activities conducted in rural areas	3.2971	1.2761	3.30 ± 1.28	0.8159
D16	Quality of sports publicity activities conducted in rural areas	3.365	1.2513	3.36 ± 1.25	0.839
D17	Protection and safeguarding of traditional rural sports	3.3808	1.2487	3.38 ± 1.25	0.8118
D18	Development of “sports + tourism” industries in rural areas	3.474	1.3026	3.47 ± 1.30	0.7861
Overall scale	D01–D18 (18 items)	3.3716			Cronbach's α = 0.9736

### 3.3 Structural adequacy and principal component extraction

The data were suitable for dimensionality reduction and structure extraction, as reported in Table 3. The Kaiser Meyer Olkin value was 0.977, indicating adequate sampling and substantial shared variance across items. Bartlett's test of sphericity was significant, chi square equals 65,339.9675 with *p* less than 0.001, confirming that the correlation matrix departs from an identity matrix and meets the basic requirements for principal component analysis. Principal component extraction showed that the first component had an eigenvalue of 12.4392 and explained 69.09 percent of total variance, far exceeding subsequent components, with PC2 and PC3 explaining only 4.27 percent and 3.55 percent, respectively. This pattern indicates a clearly dominant single dimension. Loadings for items D01 to D18 on the first component were all positive and tightly clustered between 0.2139 and 0.2465, suggesting that items contribute relatively evenly to the shared latent construct without any single item dominating or deviating. These results support the use of the first principal component score as an alternative satisfaction measure in robustness analyses to test sensitivity to outcome construction.

**Table 3:** KMO and Bartlett's test results and PCA summary for the satisfaction scale.

Panel	Statistic/ component	Result
A	KMO	0.977
A	Bartlett's χ^2^	65,339.9675
A	Bartlett's *p*-value	0
B	PC1 eigenvalue	12.4392
B	PC1 variance explained (%)	69.09
B	PC1 cumulative variance (%)	69.09
B	PC2 eigenvalue	0.7687
B	PC2 variance explained (%)	4.27
B	PC2 cumulative variance (%)	73.36
B	PC3 eigenvalue	0.6385
B	PC3 variance explained (%)	3.55
B	PC3 cumulative variance (%)	76.9
C	PC1 loadings (range)	0.2139–0.2465
C	Highest loading item	D13 quality of sports events (0.2465)
C	Lowest loading item	D05 openness of public sports facilities (0.2139)

### 3.4 Results of the multilevel linear models

**Figure 3:**
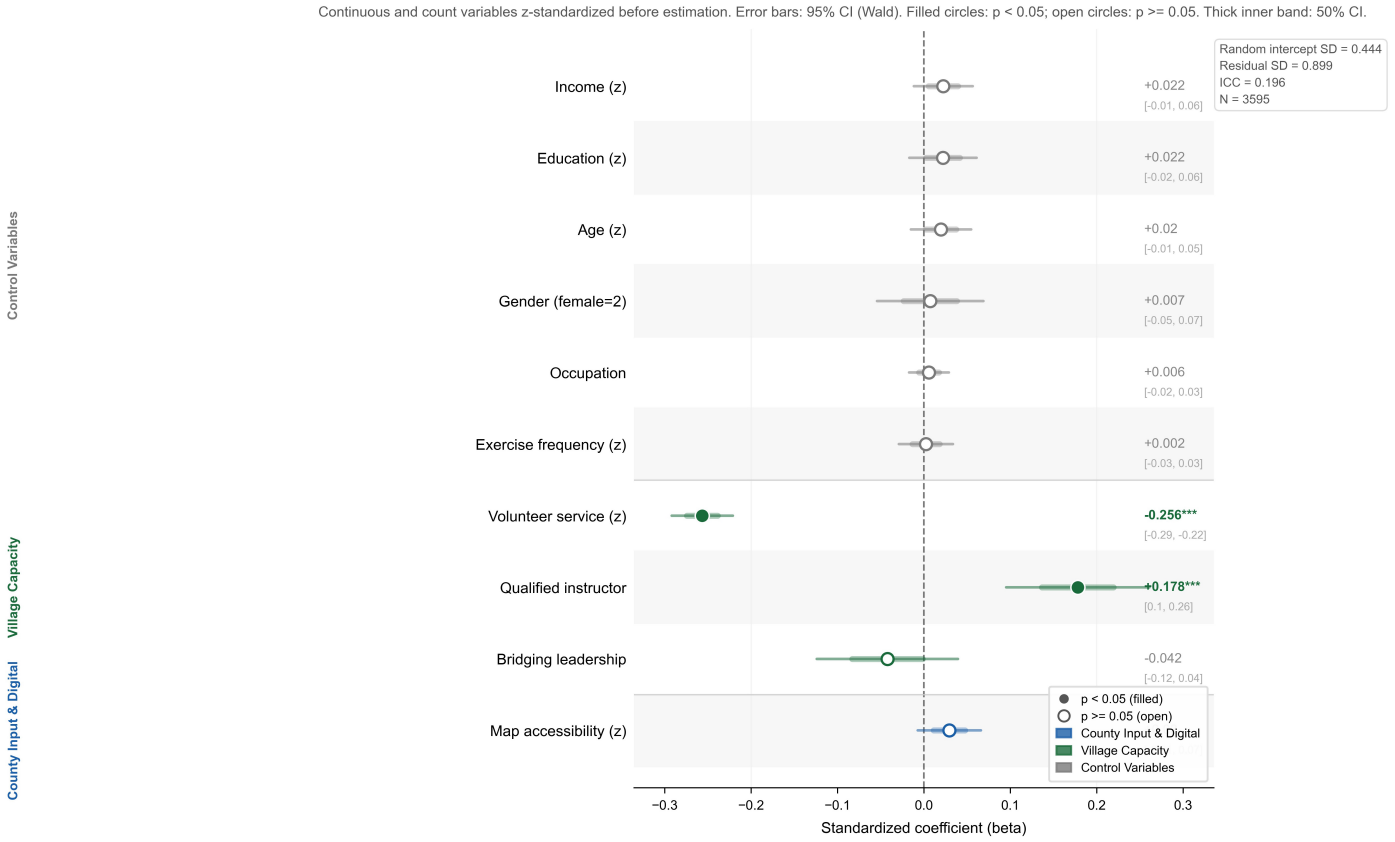
Forest plot of fixed effect estimates from the multilevel linear model with 95%.

Multilevel model estimates are reported in Table 4. The unconditional model showed pronounced village level differences. The random intercept variance was 0.2376, and the intraclass correlation coefficient was 0.2114, indicating that about 21 percent of the variation in satisfaction is attributable to differences between villages, which supports the use of multilevel modeling. After adding individual level controls in Model 1, educational attainment was positively associated with satisfaction, with beta equal to 0.0436 and *p* equal to 0.0027, whereas other demographic variables and weekly participation frequency were not significant, as shown in Figure 3. When county level digital linkage was added in Model 2, the ordered digital linkage variable C02_ord had a significant positive association with satisfaction, with beta equal to 0.1244 and *p* less than 0.001, indicating that inclusion of village facilities in the county fitness electronic map and greater usability are associated with higher satisfaction. The education effect remained weakly significant, with beta equal to 0.0301 and *p* equal to 0.0381. After introducing village organizational and human resource variables in Model 3, the presence of social sports instructors was positively associated with satisfaction, with beta equal to 0.1780 and *p* less than 0.001, while the level of volunteer service provision was negatively associated with satisfaction, with beta equal to minus 0.3134 and *p* less than 0.001. The cross domain background of the local leader was not significant. In terms of model fit, relative to earlier models, Model 3 reduced the random intercept variance from 0.2376 to 0.1971 and the residual variance from 0.8862 to 0.8090, and the intraclass correlation coefficient declined to 0.1959, suggesting that village capacity variables explain additional between village differences. Overall, digital linkage and grassroots human resources emerge as key factors associated with satisfaction differences.

**Table 4:** Random intercept multilevel linear models predicting county level urban rural sports integration satisfaction.

Section	Model	Parameter	β	SE	*p*-value	Sig.	Random-intercept var.	Residual var.	ICC	AIC	BIC	*N*
Fixed effects	M0 null model	Intercept	3.3724	0.0596	0	^***^						
Fixed effects	M1 controls	Intercept	3.1388	0.1177	0	^***^						
Fixed effects	M1 controls	A01 (gender)	−0.0059	0.0328	0.8581							
Fixed effects	M1 controls	A02 (age)	0.0173	0.0158	0.275							
Fixed effects	M1 controls	A03 (education)	0.0436	0.0145	0.0027	^**^						
Fixed effects	M1 controls	A04 (occupation)	0.0103	0.0122	0.4004							
Fixed effects	M1 controls	A05 (income)	−0.0012	0.0079	0.8786							
Fixed effects	M1 controls	B01 (exercise frequency)	0.0135	0.0143	0.3474							
Fixed effects	M2 + county-town inputs	Intercept	3.046	0.1173	0	^***^						
Fixed effects	M2 + county-town inputs	A01 (gender)	0.0084	0.0326	0.7953							
Fixed effects	M2 + county-town inputs	A02 (age)	0.0149	0.0157	0.3426							
Fixed effects	M2 + county-town inputs	A03 (education)	0.0301	0.0145	0.0381	^*^						
Fixed effects	M2 + county-town inputs	A04 (occupation)	0.0124	0.0121	0.3052							
Fixed effects	M2 + county-town inputs	A05 (income)	0.0012	0.0079	0.8737							
Fixed effects	M2 + county-town inputs	B01 (exercise frequency)	0.0108	0.0142	0.4479							
Fixed effects	M2 + county-town inputs	C02_ord (digital map linkage)	0.1244	0.0156	0	^***^						
Fixed effects	M3 + village capacity	Intercept	3.8313	0.1272	0	^***^						
Fixed effects	M3 + village capacity	A01 (gender)	0.009	0.0314	0.7756							
Fixed effects	M3 + village capacity	A02 (age)	0.0172	0.0151	0.256							
Fixed effects	M3 + village capacity	A03 (education)	0.0154	0.014	0.272							
Fixed effects	M3 + village capacity	A04 (occupation)	0.0062	0.0117	0.5936							
Fixed effects	M3 + village capacity	A05 (income)	0.0103	0.0076	0.1763							
Fixed effects	M3 + village capacity	B01 (exercise frequency)	0.0019	0.0137	0.8924							
Fixed effects	M3 + village capacity	C02_ord (digital map linkage)	0.0275	0.0173	0.1121							
Fixed effects	M3 + village capacity	Leader_bridge (bridging leader)	−0.0455	0.0422	0.2808							
Fixed effects	M3 + village capacity	Instructor (presence of instructors)	0.178	0.0431	0	^***^						
Fixed effects	M3 + village capacity	C09_ord (volunteer service level)	−0.3134	0.0219	0	^***^						
Random effects/fit	M0 null model	—					0.2376	0.8862	0.2114			3,595
Random effects/fit	M1 controls	—					0.2388	0.8846	0.2126			3,595
Random effects/fit	M2 + county-town inputs	—					0.2337	0.8695	0.2119			3,595
Random effects/fit	M3 + village capacity	—					0.1971	0.809				

^*^p < 0.05, ^**^p < 0.01, and ^***^p < 0.001.

### 3.5 Robustness checks

**Figure 4:**
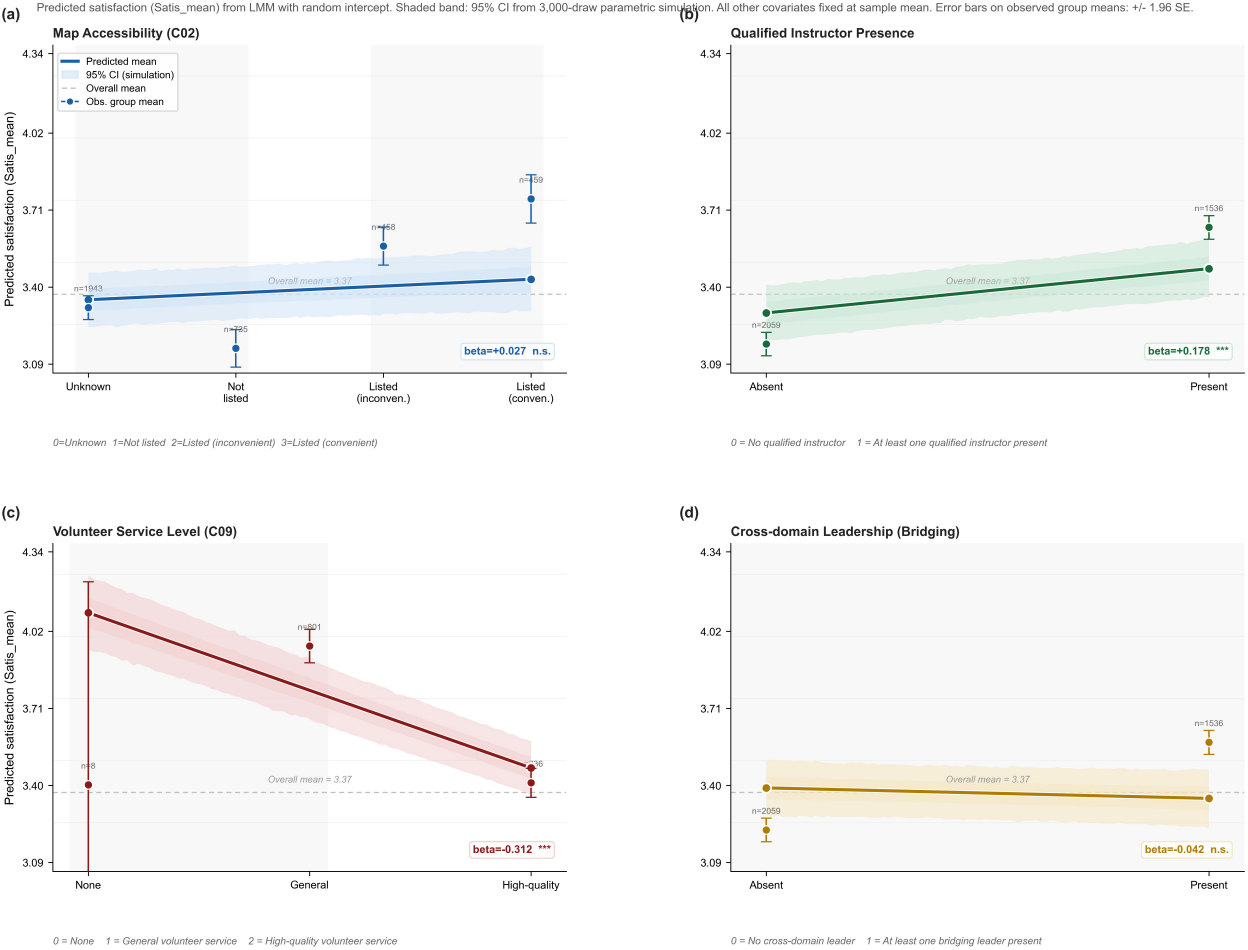
Marginal effects of digital linkage and grassroots supply capacity on predicted satisfaction.

To evaluate the robustness of the main findings, comparisons were conducted across outcome construction, estimation method, and treatment of missingness, as reported in Table 5. When the mean satisfaction score was replaced with the first principal component score, the presence of social sports instructors remained significantly positive, with beta equal to 0.5936 and *p* less than 0.001, and volunteer service level remained significantly negative, with beta equal to minus 1.0452 and *p* less than 0.001, indicating that conclusions are not driven by the specific construction of the dependent variable. Using an ordinary least squares model with cluster robust standard errors yielded consistent direction and significance for social sports instructors, beta equal to 0.2283 and *p* equal to 0.0035, and volunteer services, beta equal to minus 0.3504 and *p* less than 0.001. Under two alternative approaches to uncertain responses on C0_2_, namely treating them as missing and coding them as zero while adding an indicator for uncertainty, digital linkage remained significantly positive, with beta equal to 0.0925 and 0.0875 and *p* less than 0.01, and uncertainty itself was also associated with higher satisfaction, with beta equal to 0.1512 and *p* equal to 0.0131. By contrast, the cross domain leader background was not significant across specifications, suggesting a limited role, as illustrated in Figure 4. Taken together, the core findings are robust across multiple specifications.

**Table 5:** Robustness checks for core predictors (alternative outcomes, estimators, and missing-data handling).

Model specification	Predictor	β	SE	*p*-value	Sig.
A. LMM with PCA-based outcome (Satis_pca1)	C02_ord (digital map linkage)	0.0935	0.0577	0.1055	
	Leader_bridge (bridging leader)	−0.1512	0.1406	0.2822	
	Instructor (presence of instructors)	0.5936	0.1436	0	^***^
	C09_ord (volunteer service level)	−1.0452	0.0729	0	^***^
B. OLS with cluster-robust SE	C02_ord (digital map linkage)	−0.0013	0.0265	0.9612	
	Leader_bridge (bridging leader)	0.0338	0.0648	0.6031	
	Instructor (presence of instructors)	0.2283	0.0757	0.0035	^**^
	C09_ord (volunteer service level)	−0.3504	0.0391	0	^***^
C1. Treat C02 “unknown” as missing	C02_ord (digital map linkage)	0.0925	0.0309	0.0027	^**^
	Leader_bridge (bridging leader)	−0.0804	0.0586	0.1701	
	Instructor (presence of instructors)	0.2277	0.0612	0.0002	^***^
	C09_ord (volunteer service level)	−0.2702	0.0321	0	^***^
C2. Code C02 “unknown” as 0 and add an indicator	C02_ord (digital map linkage)	0.0875	0.0297	0.0032	^**^
	C02_unk (C02 unknown indicator)	0.1512	0.0609	0.0131	^*^
	Leader_bridge (bridging leader)	−0.041	0.0422	0.3308	
	Instructor (presence of instructors)	0.1787	0.0431	0	^***^
	C09_ord (volunteer service level)	−0.3051	0.0221	0	^***^

^*^p < 0.05; ^**^p < 0.01; ^***^p < 0.001.

LMM, linear mixed-effects model; OLS, ordinary least squares with cluster-robust standard errors.

## 4 Discussion

At the county level, urban rural sports integration is reflected in several service links that residents can directly experience, including access to venues and opening policies, county to village support for events and activities, fitness guidance and training provision, information access through tools such as the fitness electronic map, and village level organization and volunteer support. The satisfaction scale used in this study was designed to capture these links from the perspective of rural residents.

County level urban rural sports integration has become a central issue for improving equity in rural public sport services (16, 17). However, prior research has largely emphasized provision scale or participation rates, leaving limited direct evidence on rural residents integration satisfaction and the sources of its village level variation. The roles of digital linkage and grassroots human resource supply also remain insufficiently tested. Using data from 3,595 rural residents nationwide, this study finds a moderately high overall satisfaction level, with a mean of 3.37. At the item level, ratings were highest for the opening of county seat sport venues at free or low cost and lowest for the development of the fitness electronic map. Principal component analysis indicates a clearly dominant single dimension, supporting the use of a composite index to represent overall satisfaction. Multilevel models show substantial village level heterogeneity and limited explanatory power of individual characteristics. Digital linkage is positively associated with satisfaction, but the association weakens after village supply capacity is included. In the final model, grassroots human resource supply provides stronger explanatory leverage. The presence of social sports instructors is consistently positively related to satisfaction, whereas volunteer service level shows a consistent negative association with satisfaction. Robustness checks using alternative outcomes, alternative estimators, and different missing data treatments yield consistent conclusions, indicating that the main findings are highly robust.

Rural residents overall perceptions of county level urban rural sports integration are generally positive, but perceived gains across different elements are uneven. Services that directly improve day to day exercise experience are more likely to translate into higher evaluations. By contrast, digital components represented by information platforms and online services continue to show a mismatch between construction and actual use (18). This pattern suggests a gradient between visible provision and less visible governance capacity. Satisfaction exhibits strong internal coherence and aggregation, implying that residents tend to form an overall impression of integration performance (19). A composite index can therefore capture the general evaluation in a stable way, while item level differences are more informative for diagnosing weaknesses and locating directions for improvement.

Village context plays a prominent role. Satisfaction is not primarily driven by differences in individual attributes, but depends more on the visible performance of grassroots supply conditions and governance capacity (20). This implies that socioeconomic disparities may affect satisfaction mainly through contextual differences in service accessibility and supply quality rather than through direct effects on evaluation. Grassroots human resource supply shows stable associations in the models, highlighting that organized provision and competent instruction are critical for improving perceived gains (21, 22). The volunteer service variable remains significant across specifications, indicating that its operational quality, continuity, and fit with local needs may directly shape residents experience, and that effectiveness cannot be judged simply by whether services are offered (23, 24). In contrast, a cross domain background of local leaders does not show a stable effect, suggesting that personal labels cannot substitute for institutionalized supply (25, 26). The magnitude of the digital linkage association varies across models, implying that the value of digital linkage likely depends on offline supply capacity, information reach, and actual usability. Policy priorities therefore need to move beyond platform deployment toward making services usable, routinely used, and reliably accessible.

Much of the existing literature centers on rural residents sport participation and emphasizes participation environments and supply conditions, often focusing on public sport facilities and community sport provision and their links to participation opportunities and health outcomes (27, 28). Another strand focuses on public service satisfaction and documents urban rural gaps and regional patterns, frequently noting that rural residents assign lower ratings to supportive and managerial services (24) d focuses on public service satisfaction and documents urban rural gaps and regional patterns, frequently noting that rural residents assign lower ratings to supportive and managerial services (29). Within this context, relatively few studies treat satisfaction with county level urban rural sports integration as the primary outcome, and even fewer integrate digital linkage and grassroots human resources within a single explanatory framework. This study begins from the perspective of rural residents, develops and validates the measurement structure of integration satisfaction, and then re situates individual evaluations within village context using a national sample. In doing so, it avoids reducing integration performance to personal preference differences and addresses more directly how integration is perceived in governance practice.

Many satisfaction studies argue that urban rural disparities are more closely tied to deficiencies in supportive services rather than to whether facilities are sufficient or whether prices are high (30). The present findings point in the same direction, with perceived shortcomings concentrated in information acquisition and the service chain. This indicates that once county level integration enters a stage of moving from existence to quality, institutional arrangements and service capacity can matter more for perceived gains than sheer facility counts (31). Unlike studies that emphasize individual socioeconomic status, the effects of individual level variables in this study shrink after village supply capacity is included. A plausible interpretation is that socioeconomic differences are often amplified or buffered through village level supply conditions and service accessibility (32). This aligns with repeated conclusions in policy evaluation research regarding mismatches between supply and demand and weak service reach. Another notable result is that a cross domain leadership background does not provide stable explanatory power. Who leads does not automatically translate into sustained, professional, and accessible services. Whether governance resources are institutionalized and routinized is likely more consequential.

The nontrivial share of variance at the village level suggests that satisfaction with urban rural sports integration is best understood as a perception of grassroots governance performance. Residents evaluate not only their personal circumstances but also whether villages have stable organizational operations, sustained human resource provision, and a service chain that functions. The strong positive association of social sports instructors supports this view. Instructors serve as a key implementation resource in grassroots sport services (13, 33). They turn facilities and events into concrete opportunities to participate, increase the frequency of organized activities, lower participation barriers, and improve experience through more professional guidance, thereby raising overall evaluations. The stable association between volunteer services and satisfaction also deserves attention (34). Volunteer services are not merely ornamental. They enter residents evaluative frame. The coding direction of this variable should be clearly stated so that readers do not mechanically interpret the coefficient sign as implying that stronger volunteer services reduce satisfaction.

The attenuation of the digital linkage effect after introducing village capacity variables suggests that the electronic map functions more as an amplifier than as a substitute. Whether the platform creates value depends on whether offline provision can support use, whether information dissemination is adequate, and whether the system is truly user friendly. Prior research repeatedly points to limited facility openness and barriers to service access, which provide an institutional backdrop for the reality of having a platform that is difficult to use (35). Individuals with limited awareness of digital services may not lower their evaluations due to frustration in use. They may instead form relatively moderate overall judgments based on visible offline provision. Work on digital governance, including discussions of regional disparities and the digital divide, also indicates that reach and usability must be addressed together. The non-significance of leader background further indicates that symbolic mobilization cannot replace stable provision. Where a leader comes from does not necessarily translate into sustained, professional, and accessible services (36). Any potential influence may also be absorbed by more proximal supply variables such as instructors and volunteer services.

To clarify how county level urban rural sports integration is implemented in practice, Supplementary Figure S2 summarizes the governance and service delivery chain and links each component to the key variables examined in this study. Our study grounds county level urban rural sports integration in service performance that residents can directly perceive, rather than remaining at the level of macro narrative (37). It brings digital linkage, grassroots human resource supply, and village contextual differences into a single explanatory framework, and makes the shift in public sport services from supply side construction to experience oriented governance empirically testable. The findings suggest that counties should advance integration by strengthening both mechanisms and capacity. Coordination of events and activities between county seats and villages needs to become routine, with stable channels for organizational input. County fiscal investment and social capital participation should be combined so that funding is both more stable and more diversified. Development of the fitness facility electronic map should not focus only on inclusion coverage. The platform needs to be easy to search, easy to use, and regularly updated. Villages should address deficits in sport organizations and instructor teams and establish effective linkages with volunteer service systems associated with the New Era Civilization Practice Centers, ensuring that service quality and continuity are embedded in day to day operations. Efforts to document and safeguard traditional sports should also be advanced in coordination with sport plus folk culture initiatives so that county level integration rests on a stronger sociocultural foundation.

Several limitations should be noted. First, the cross sectional design limits causal inference, particularly because service provision and satisfaction may influence each other over time. The estimated effects should therefore be interpreted as associations. Second, most variables were self reported, which may introduce recall error and social desirability bias. The presence of uncertain responses also suggests that uneven access to information may shape how services are evaluated. Third, we did not have county administrative data on facility density, opening hours, fiscal inputs, or instructor staffing, which may constrain generalizability and leave residual confounding. Future work should link resident surveys with administrative records, follow changes longitudinally or through before after evaluations of policy pilots, and apply phased implementation or quasi experimental designs to strengthen identification of digital linkage and grassroots capacity effects.

## 5 Conclusions

This study uses satisfaction with county level urban rural sports integration as a composite performance indicator of residents perceived gains. Using a national sample of 3,595 rural residents, we operationalized service experience through a validated 18 item scale and examined village clustered variation with a random intercept multilevel model. Overall satisfaction was moderately high, the scale showed strong internal consistency and a dominant single dimension, and a substantial share of variance was attributable to villages. Individual demographic characteristics and participation patterns explained limited variation. Digital linkage through the county fitness electronic map was positively related to satisfaction, but the association attenuated after accounting for village supply capacity, suggesting that digital tools depend on offline provision and practical usability. Grassroots human resource supply showed stronger and more stable associations. The presence of social sports instructors was consistently linked to higher satisfaction, whereas volunteer service level was consistently linked to lower satisfaction. A cross domain leadership background did not show a stable effect. Robustness checks across alternative outcomes, estimators, and handling of uncertain responses supported the main conclusions. These findings indicate that county governance should prioritize usability and lived experience. Practical priorities include strengthening instructor staffing and routine organizational operations, improving the quality and institutionalization of volunteer services, and maintaining a fitness electronic map that is easy to access, regularly updated, and aligned with offline service delivery.

## Data Availability

The raw data supporting the conclusions of this article will be made available by the authors, without undue reservation.

## Supplementary material

Supplementary Figure S1 Provincial boundaries of China showing the four macro regions and the sampled provinces. Provinces were grouped into Eastern, Central, Western, and Northeastern China following the National Bureau of Statistics regional classification. Sampled provinces are highlighted with bold outlines. Boundary data were obtained from GADM version 4.1.

Supplementary Figure S2 Conceptual workflow of county level urban rural sports integration governance and service delivery. The diagram outlines county inputs, township coordination, village level delivery, human resource provision, and digital support through the fitness e map, together with a feedback loop for service improvement.
