# Supplementary figures and images for: Satisfaction with urban rural sports integration at the county level in China and its determinants
Source: Front Public Health. 2026 Apr 30;14:1821897. doi: 10.3389/fpubh.2026.1821897 (PMC13171499; doi:10.3389/fpubh.2026.1821897)

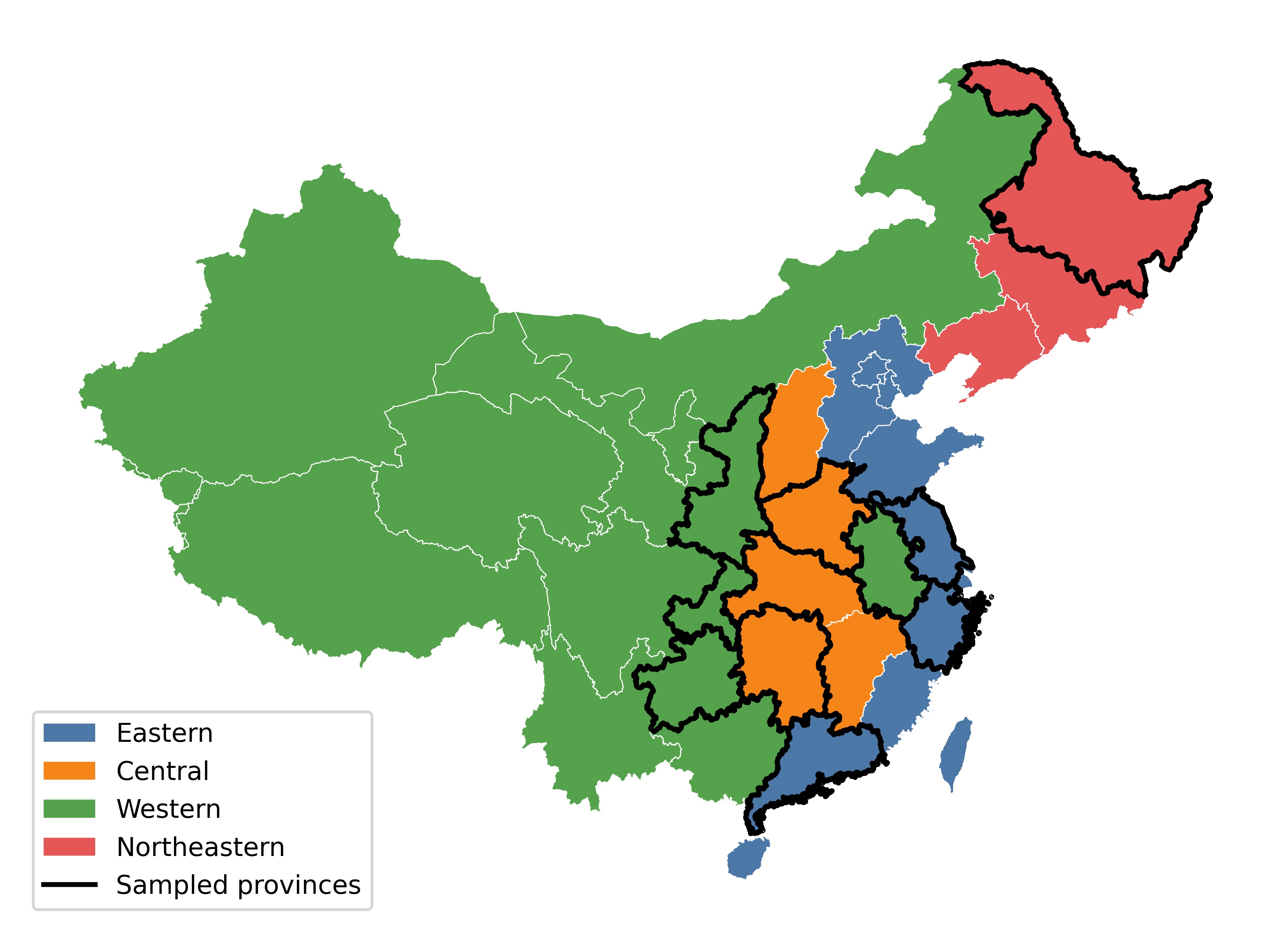

Supplement: Supplementary Figure S1 — Provincial boundaries of China showing the four macro regions and the sampled provinces. Provinces were grouped into Eastern, Central, Western, and Northeastern China following the National Bureau of Statistics regional classification. Sampled provinces are highlighted with bold outlines. Boundary data were obtained from GADM version 4.1. [file Image_1.JPEG]

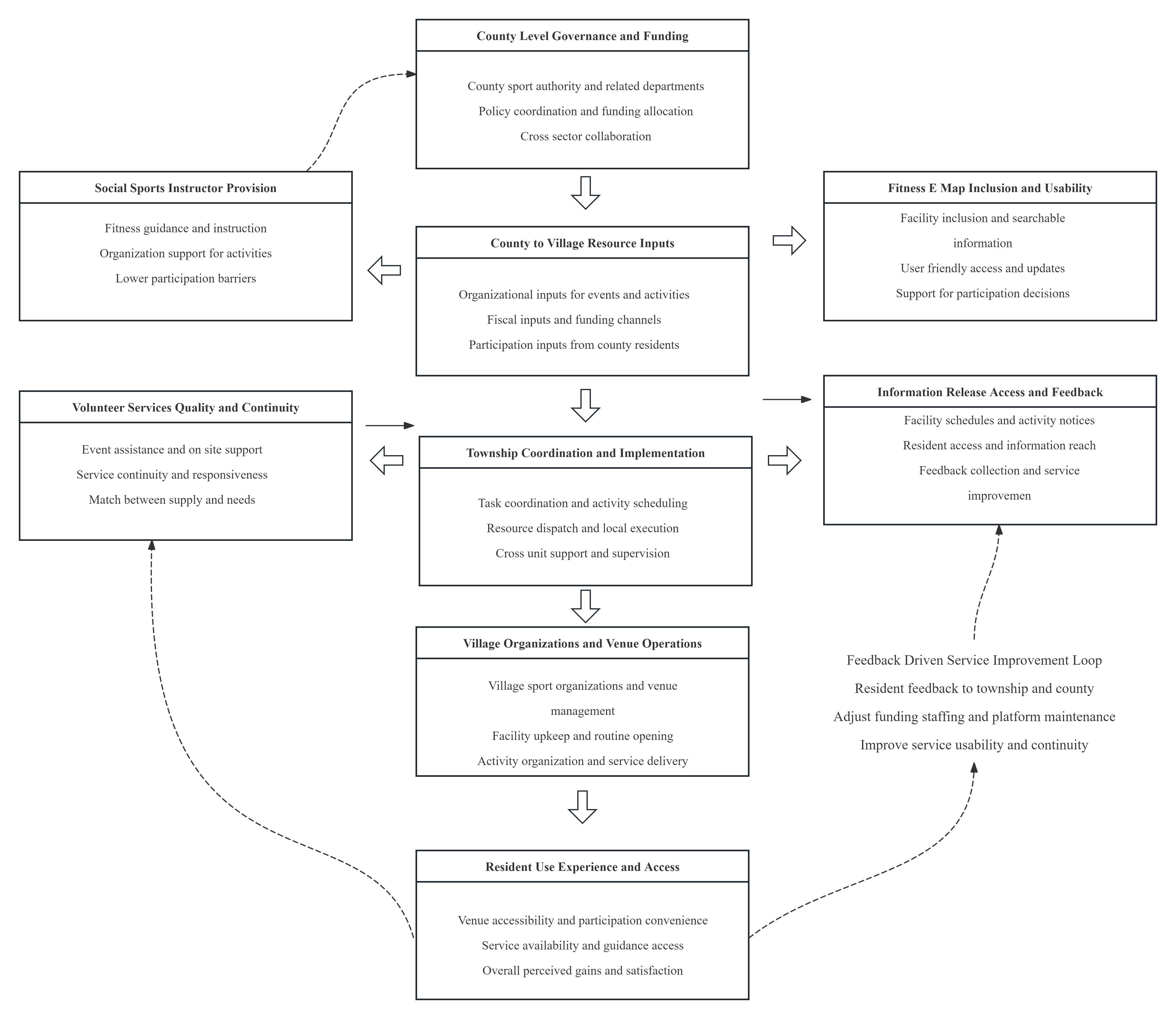

Supplement: Supplementary Figure S2 — Conceptual workflow of county level urban rural sports integration governance and service delivery. The diagram outlines county inputs, township coordination, village level delivery, human resource provision, and digital support through the fitness e map, together with a feedback loop for service improvement. [file Image_2.JPEG]
